# Regorafenib and glioblastoma: a literature review of preclinical studies, molecular mechanisms and clinical effectiveness

**DOI:** 10.1017/erm.2024.8

**Published:** 2024-04-02

**Authors:** Maria Patrizia Mongiardi, Roberto Pallini, Quintino Giorgio D'Alessandris, Andrea Levi, Maria Laura Falchetti

**Affiliations:** 1 Institute of Biochemistry and Cell Biology, IBBC-CNR, Monterotondo, Rome, Italy; 2 Department of Neuroscience, Neurosurgery Section, Università Cattolica del Sacro Cuore, Rome, Italy; 3 Department of Neurosurgery, Fondazione Policlinico Universitario A. Gemelli IRCCS, Rome, Italy

**Keywords:** glioblastoma IDH-wild type, glioma stem cells, multikinase inhibitor, regorafenib, therapy

## Abstract

Glioblastoma IDH wild type (GBM) is a very aggressive brain tumour, characterised by an infiltrative growth pattern and by a prominent neoangiogenesis. Its prognosis is unfortunately dismal, and the median overall survival of GBM patients is short (15 months). Clinical management is based on bulk tumour removal and standard chemoradiation with the alkylating drug temozolomide, but the tumour invariably recurs leading to patient's death. Clinical options for GBM patients remained unaltered for almost two decades until the encouraging results obtained by the phase II REGOMA trial allowed the introduction of the multikinase inhibitor regorafenib as a preferred regimen in relapsed GBM treatment by the National Comprehensive Cancer Network (NCCN) 2020 Guideline. Regorafenib, a sorafenib derivative, targets kinases associated with angiogenesis (VEGFR 1-3), as well as oncogenesis (c-KIT, RET, FGFR) and stromal kinases (FGFR, PDGFR-b). It was already approved for metastatic colorectal cancers and hepatocellular carcinomas. The aim of the present review is to focus on both the molecular and clinical knowledge collected in these first three years of regorafenib use in GBM.

## Graphical abstract

**Figure:**
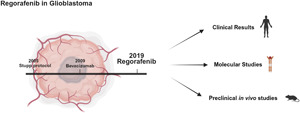

## Introduction

Glioblastoma IDH wild type (GBM), previously named glioblastoma multiforme due to its huge intra- and inter-tumour heterogeneity, is the most aggressive brain tumour in the adult. In the 5th edition (2021) of the WHO classification of CNS tumours (Ref. 1), GBM have been defined as diffuse astrocytic tumours in adults that must be IDH-wild type and are a totally separate diagnosis from astrocytoma, IDH-mutant grade 2, 3 or 4. GBM is defined by a series of peculiarities. Histologically, it is distinguished by rapid mitotic activity and microvascular proliferation or necrosis. Molecularly, it is characterised by the presence of TERT (telomerase reverse transcriptase) promoter mutations, EGFR gene amplification or copy number changes (Refs 2, 3), and by lack of IDH1 (isocitrate dehydrogenase), IDH2 and histone H3 mutations. In addition, GBM might further be classified into four subtypes: proneural, neural, mesenchymal and classical (Ref. 4). The subtype can be associated with progression. Clinical outcome of GBM is dismal, with a median survival at diagnosis of about 15 months and a 5-year relative survival rate of only 6.8% (Refs 5, 6). Current standard of care is maximal safe surgical resection followed by radiation and chemotherapy with the alkylating agent temozolomide (TMZ) (Ref. 5). Despite this, because of its highly infiltrative nature, the tumour invariably recurs. Therapeutic options at recurrence are scarce, with lomustine, which is the standard of care, offering only few months survival (Ref. 7).

**Figure 1.**
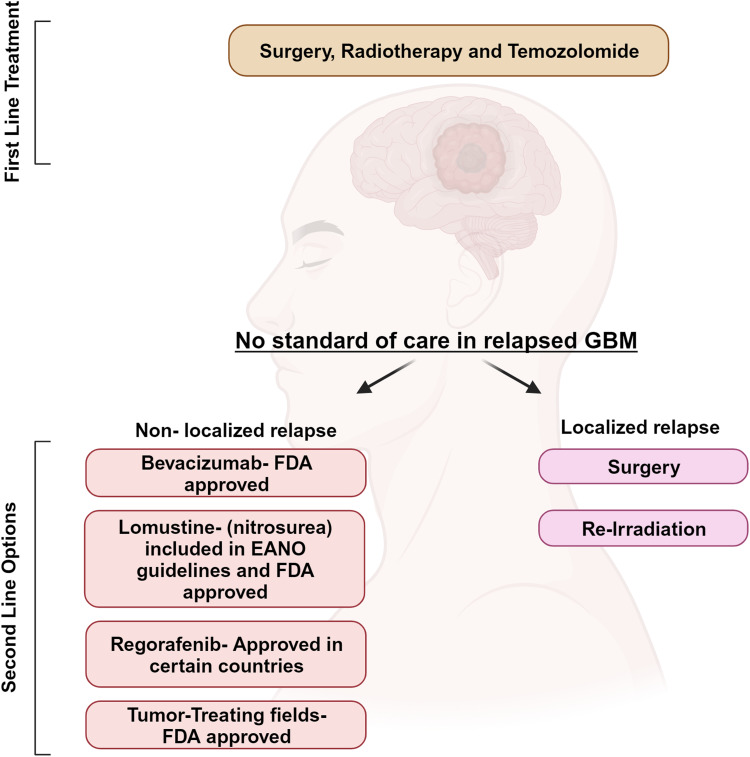
Current therapeutic approach for GBM patients. First-line treatment for GBM patients is based on surgery and adjuvant chemoradiation with the alkylating agent temozolomide. At tumour relapse, second-line treatment is lomustine, the humanised monoclonal antibody bevacizumab, which targets vascular endothelial growth factor (approved limiting to US, Japan and China) and the recently approved regorafenib. Created with Biorender.com.

GBM is paradigmatic in its ability to induce neoangiogenesis and its growth relies on the induction of massive angiogenesis for growth and progression (Ref. 8). This is the reason why the most recent therapeutic attempts have been primarily focused on neoangiogenesis inhibition. Different strategies to inhibit vascular endothelial growth factor (VEGF) or its receptors (VEGFRs) have been developed, but the overall result of this approach has substantially disappointed the expectation (Ref. 9), and no significant improvement in the management of GBM patients has been reached for more than 20 years. A milestone in the history of GBM treatment studies is represented by the REGOMA trial in 2019, which paved the way for regorafenib introduction in clinical practice for this disease. In the USA, regorafenib has been included since 2020 in the National Comprehensive Cancer Network (NCCN) Guidelines, and in Italy, in the same year, it was approved for recurrent GBM treatment by the Italian Agency of Medicine (AIFA) (Fig. 1). REGOMA was a randomised phase II multicentre clinical trial conducted on relapsed GBM patients that compared regorafenib effectiveness with a control arm, where patients were treated by lomustine. In this study, it has been observed that relapsed GBM patients treated with regorafenib experienced an increase in overall survival (OS) when compared to the control arm (Ref. 10). For this reason, regorafenib is considered the first drug in the last 20 years to demonstrate efficacy in GBM therapy. However, it is important to highlight that together with enthusiasm for REGOMA trial, some concerns have been expressed and discussed about (i) data obtained in control arm, characterised by a poorer outcome than other studies; (ii) the presence of IDH-mutant patients in regorafenib treated arm; (iii) the lack of centralised pathology and molecular review (Ref. 11).

Three years later, this review assesses the state of the art, on both molecular and clinical sides, of regorafenib use in GBM.

### Regorafenib

**Figure 2.**
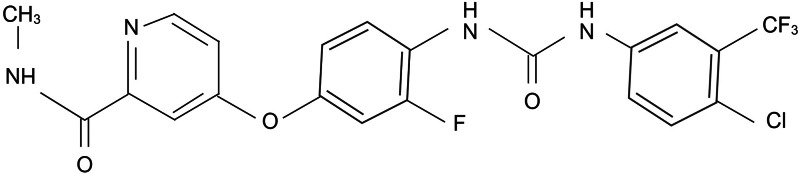
Molecular structure of regorafenib. Created with Biorender.com

Regorafenib (BAY 73-4506) is an oral multikinase inhibitor of angiogenic, stromal and oncogenic receptor tyrosine kinases (RTKs). Its molecular structure is similar to the one of sorafenib (Fig. 2), although regorafenib is characterised by enhanced pharmacologic activity when compared to sorafenib (Refs 12, 13, 14).

**Figure 3.**
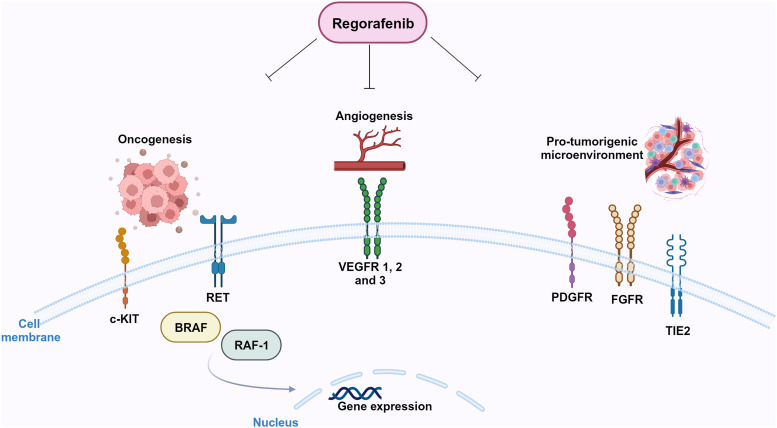
Molecular targets of regorafenib. Regorafenib targets stromal (FGFR, PDGFR), angiogenic (VEGFRs, Tie-2) and oncogenic kinases (RET, KIT). Created with Biorender.com.

In preclinical models, it was firstly shown that regorafenib is able to target RTKs (Ref. 15): kinases involved in tumour angiogenesis (VEGFR1-3) as well as in oncogenesis processes (KIT, RET, RAF-1, BRAF and BRAFV600E) and tumour microenvironment (PDGFR and FGFR) (Refs 15, 16, 17) can be inhibited by regorafenib (Fig. 3). Regorafenib targets immunity as well, by inhibiting colony-stimulating factor-1 receptor, therefore impairing macrophage differentiation and survival, and causes reduction in tumour-infiltrating macrophages (Refs 18, 19). Xenograft models of different tumours (lung, melanoma, pancreatic and ovarian tumours) were used to study the anti-tumour potential of regorafenib, and its anti-angiogenic effect was observed in rat GBM xenograft model (Ref. 15). In 2012, 2013 and 2017, on the basis of encouraging clinical trials, FDA approved the clinical use of regorafenib for advanced colorectal cancer, advanced gastrointestinal stromal tumours and advanced hepatocellular carcinoma, respectively (Refs 20, 21, 22).

### Clinical results of regorafenib on recurrent GBM

GBM relapse is one of the main challenges in neuro-oncology. However, as mentioned, REGOMA trial showed that regorafenib significantly improves OS when compared to lomustine (7.4 versus 5.6 months, respectively) (Ref. 10). Regoma was conducted in Italy on 119 relapsed GBM patients, randomly assigned to regorafenib arm (59 patients) or lomustine arm (60 patients). The gender distribution was 69% males and 31% females in the regorafenib arm, and 72% males and 28% females in the lomustine arm. Forty-nine per cent of patients had methylated MGMT and 95% were IDH wild type. According to ClinicalTrials.gov, there are currently several ongoing clinical trials on regorafenib in recurrent GBM and one on parallel groups of patients affected by relapsed or metastatic cancers. Table 1 summarises their main features. ‘Glioblastoma (GBM) adaptive, global, innovative learning environment’ (GBM AGILE) is currently active and recruiting. It is a wide international phase II/III response adaptive randomisation platform trial designed to evaluate multiple therapies in newly diagnosed (ND) and relapsed GBM. The study, which plans to enrol 1030 participants, focuses on the identification of new therapies for GBM and meets effective therapies with specific patients' subgroups. The AGILE protocol allows simultaneous evaluation of drug combinations from different companies. The primary endpoint of the study is OS. Finally, a phase I ongoing Italian trial is investigating the possibility of using regorafenib in ND, MGMT promoter methylated GBM, IDH wild type, in combination with the currently used chemoradiation therapy (Ref. 23). The side effects reported in REGOMA reflect what was observed for hepatocellular carcinoma patients: grade 3 and 4 clinical adverse events such as hypertension, hand-foot skin reaction, fatigue and diarrhoea (Refs 22, 24). REGOMA team reported that the frequency of undesired side effects was higher in regorafenib-treated patients than in lomustine-treated (control) patients (Ref. 10). Nevertheless, regorafenib did not impair patients' quality of life compared to the control group (Ref. 25). In order to explore the possibility of scaling down regorafenib dosage to set up the best cost/benefit ratio, Rudà *et al*. (Ref. 26) evaluated in a real life study the efficacy and tolerability of a lower intensity regimen. Interestingly, authors reported no loss of efficacy in terms of both PFS and OS. In parallel, adverse effects of regorafenib therapy were reduced in terms of incidence. The cohort of patients enrolled in this study had a median age higher than that of the REGOMA trial (60 versus 54.8 years, respectively), suggesting that age might represent a predictive factor for susceptibility to regorafenib. The issue of the role of age on response to regorafenib was analysed by Fasano *et al.* too (Ref. 27). They reported the result of a retrospective study on recurrent GBM patients treated with regorafenib. The primary endpoint of the study was OS, with PSF, objective response rate and disease control being secondary endpoints. In the frame of this study, age did not significantly affect median PFS, while MGMT methylation status positively correlated with median OS. The point that patients' age is not a parameter which affects response to regorafenib is important, since it supports the idea that treatment choice should not vary in elderly patients. Further studies are needed to address this issue.

**Table 1.** Active clinical trials on regorafenib therapy in GBM

Status	Study title	Phase
Active, not recruiting	Regorafenib in bevacizumab refractory recurrent glioblastoma	II
Active, recruiting	Regorafenib in patients with relapsed glioblastoma. IOV-GB-1-2020 REGOMA-OSS	Not applicable
Active, recruiting	Biomolecular analysis for predicting response to regorafenib (RegoRec)	Not applicable
Active, not recruiting	A trial to learn whether regorafenib in combination with nivolumab can improve tumor responses and how safe it is for participants with solid tumors^a^	II
Active, recruiting	Glioblastoma (GBM) adaptive, global, innovative learning environment (GBM AGILE)	II/III

a Disease: solid tumours, including GBM

A detailed assessment of regorafenib safety after its widespread use in recurrent GBM is yet to be performed.

We have recently treated with regorafenib 30 patients with recurrent GBM, IDH wild type, after standard radiotherapy *plus* concomitant and adjuvant TMZ (Ref. 28). The mean age was 58.4 years with Karnofsky performance status ⩾70 in all cases. Median follow-up was 20 months. The cohort had a median PFS of 3 months. Twenty-nine per cent of regorafenib-treated patients had a 6 months PFS. Median OS was 7.5 months. We calculated PFS and OS from regorafenib start to progression of disease and patient's death, respectively. Remarkably, median PFS and median OS in our study are highly comparable to those reported in the REGOMA trial (Ref. 10). Further studies do not specifically address the effect of regorafenib on patients' survival, rather they focus on the identification of predictive factors allowing the selection of those patients with the highest possibilities to positively respond to regorafenib.

Response to treatment in cancer patients still remains an unsolved issue. In the clinical practice, the response to treatment is assessed on magnetic resonance imaging (MRI) using the RANO criteria (Ref. 29). According to RANO, patients response to therapies might be classified as partial response (PR), requiring ⩾50% reduction of contrast enhancement without increase in FLAIR alterations; progressive disease (PD), with ⩾25% increase of contrast enhancement or increase in FLAIR alterations; stable disease (SD) does not qualify either for PR or for PD. In our study (Ref. 28), we record PR, SD and PD in 18.5, 29.6 and 25.9% of cases, respectively. In seven other cases (25.9%), MRI exams were not done at clinical progression. However, a recent study suggests caution in assessing treatment response based on contrast-enhanced MRI (Ref. 30). Werner *et al.* reported on ill-defined MRI findings of recurrent IDH-wild type GBM treated with regorafenib (Ref. 30). During treatment, imaging showed correlates of extensive necrosis with contrast-enhancing rim on serial MRI. Also, restricted diffusion MRI combined with partially low and decreasing apparent diffusion coefficient values suggested increased cellularity. Conversely, the relative cerebral blood volume on perfusion weighted MRI was equal to zero. At the end, stereotactic biopsy of tissue with MRI changes identified reactive histology compatible with necrotic tissue.

### Molecular studies on GBM cells response to regorafenib

#### Regorafenib and autophagy

Although regorafenib has been introduced in the clinical management of relapsed GBM patients, the molecular basis of tumour response to regorafenib are still poorly understood. It has been shown that regorafenib induces lethal autophagy (Ref. 31). Autophagy plays a key role in many diseases such as cancer. It allows to degrade misfolded proteins or damaged organelles, in a complex biological process that involves the formation of autophagosomes and autolysosomes organelles, regulated by complex mechanisms that have not been completely described yet. Autophagy response is induced by different stress stimuli and preserves cellular homeostasis under physiological conditions (Ref. 32). It has opposing, context-dependent roles in cancer, and interventions to both stimulate and inhibit autophagy have been proposed as cancer therapies (for a review, see (Ref. 33)). The pro-autophagy activity of several chemotherapeutic drugs has been extensively reported in different tumours (Ref. 34). Autophagy has been reported in GBM in response to TMZ, and it has been hypothesised that it confers a compromised therapeutic response (Refs 35, 36). Regorafenib-dependent autophagy in GBM, as reported in (Ref. 31), is one of the main causes of growth arrest of GBM cells. It has been observed that regorafenib binds and stabilises PSAT1 (phosphoserine aminotransferase 1). This stabilisation leads to PRKAA (protein kinase AMP-activated catalytic subunit alpha) activation and consequent autophagy induction. At the same time, PSAT1 inhibits RAB11A, a key protein for autophagosome–lysosome fusion, and consequently, causes an accumulation of autophagosomes inside the cells, that induce cell death. Regorafenib might induce cell death in GBM, either established cell lines and GSCs via induction of apoptosis (Ref. 37). In line with this finding, we demonstrated apoptotic induction following regorafenib exposure, but the percentage of apoptotic cells was low, suggesting that this is not the main route of regorafenib-triggered death induction (Ref. 38).

#### Regorafenib impact on glioma stem cells

A significant impact of regorafenib on the glioma stem cell (GSC) sub-population was also reported (Ref. 39). GSCs represent the cellular fraction responsible for GBM recurrence, and are the cellular subpopulation which is most resistant to anticancer drugs (Ref. 40). As mentioned before, aberrant and massive neoangiogenesis is a key feature of GBM tumours. Neoangiogenesis in GBM relies on different mechanisms: vascular co-option, vascular mimicry, transdifferentiation (Ref. 41). Endothelial transdifferentiation of GSC is essential for tumour growth (Refs 42, 43, 44) and this phenomenon is enhanced upon irradiation (Ref. 45). A recent paper specifically addressed the issue of regorafenib impact on GSCs differentiation towards the endothelial phenotype (Ref. 39). Authors treated two GSC cell lines (derived from a classical and from a proneural GBM, respectively) with regorafenib, either in vitro or in mouse xenografts, and obtained a consistent reduction of CD31-expressing cells, as well as a general, dose-dependent reduction of GSCs pro-angiogenic ability. Although it is expected that regorafenib, which targets the VEGF/VEGFR pathway, inhibits angiogenesis, the impairment of GSC-treated endothelial transdifferentiation ability was not expected. The mechanisms driving GSC transdifferentiation are still poorly understood. Some studies demonstrated that the VEGF signalling pathway is involved, whereas others showed that transdifferentiation is VEGF-independent, a data which could account for GBM resistance to anti-angiogenic therapies (Refs 43, 44, 46, 47). In the case of regorafenib, we can hypothesise that the observed reduction of transdifferentiation capacity might be ascribed to the wide anti-kinase inhibitory activity of the drug. Another major effect reported by the same team was the regorafenib effectiveness to impair GSC ability to develop neurospheres, a key stem cell feature (Ref. 39). We recently confirmed this observation in our GSC lines and with different experimental settings (Ref. 38). In detail, we used two different GSC cell lines (GSC#1 and GSC#83, derived from proneural and mesenchymal tumour subtype, respectively), treated with 7.5 μM regorafenib, a condition that reflects the reported patients' plasma regorafenib levels. We demonstrated that the previous observation can be extended to other GSC cellular models, therefore supporting the idea that regorafenib might affect the stemness properties of GSC. In the same study, we confirmed in 2D cell cultures and in 3D tumour spheroids that regorafenib strongly impairs GBM viability. However, by gene expression analysis on a group of epithelial to mesenchymal transition-related genes, we observed the transcriptional modulation of this pathway. This ability of regorafenib to induce a migratory phenotype on surviving cells has been also observed on 3D cultures in which invading cells migrate through matrix. Although these data deserve further investigation, the possibility that regorafenib administration might trigger an infiltrative shift must be taken into careful consideration. Induction of pro-invasive phenotype by drugs targeting the angiogenic pathway in GBM has been described for the humanised monoclonal anti-VEGF antibody bevacizumab (Refs 9, 48, 49), a feature which is responsible for the overall disappointing results of this molecule for GBM therapy. Strikingly, we described a significant upregulation of TEM7 gene mRNA, a gene which plays a crucial role in the bevacizumab-mediated infiltrative shift of GBM in vitro and in animal models (Ref. 48). Furthermore, our in vitro data are in some way supported by clinical evidences. A recent paper reported about a pseudoresponse (as detected by MRI) in a GBM patient treated with regorafenib at tumour relapse (Ref. 50). The phenomenon of pseudoresponse is a consequence of the use of anti-angiogenic drugs that maintains an undisrupted blood–brain barrier (BBB), leading to a decrease of tumour enhancement even in rapidly growing tumours (Ref. 49). In other words, BBB stabilisation following anti-angiogenic drugs administration results in a decrease in tumour-enhancing T1-weighted images, an infiltrative non-enhancing relapse and worsening of the clinical picture. The same was reported for regorafenib (Ref. 50). Gatto *et al.* describe the decrease in tumour enhancement according to MacDonald's criteria, which was not accompanied by a significant antitumour effect (Ref. 51). The MRI pattern in part overlapped with that extensively observed in relapsed GBM patients treated with bevacizumab.

#### Predictive factors for regorafenib response

The first *omic* study conducted on GBM patients treated with regorafenib was developed on patients enrolled in the REGOMA trial, developed in 10 Italian centres (Ref. 10). It was performed with the goal of identifying a signature potentially predictive of responsiveness to regorafenib (Ref. 52). Santangelo *et al.* analysed genome-wide transcriptome and miRNome in tumour specimens from primary surgery and correlated their expression levels with the OS and PFS in the two arms of treatment, regorafenib and lomustine. Although this approach does not include tumour specimens from regorafenib-treated patients, since it was carried on tumour samples from primary surgery, that is, before regorafenib administration, it draws a correlation between drug responsiveness and patients' transcriptome/miRNome. The study identifies molecular traits which correlate with an increased susceptibility to regorafenib. A mini signature composed by two upregulated genes, HIF-1*α* (hypoxia inducible factor 1*α*) and CDKN1A (cyclin dependent kinase inhibitor 1A), and three downregulated miRNAs, miR-93-5p, miR-3607-3p and miR-301a-3p, was identified which correlated with better prognosis upon regorafenib treatment. The study is descriptive, notwithstanding it is a first insight in the direction of GBM patients' stratification, responders versus non-responders, supporting the clinical decision about regorafenib use.

Recently, we published a second *omic* study (Ref. 28). The goal of the study was to characterise molecular predictive factors for response to regorafenib in GBM patients. We analysed a cohort of 30 patients with relapsed GBM, IDH-wild type, who were treated with regorafenib at tumour relapse. We performed NGS sequencing on FFPE tumour specimens from first surgery, finding that 18% of the cases presented mutation in the EGFR and in the mitogen-activated protein-kinase (MAPK) pathway. Of note, a correlation was observed between MAPK pathway mutation and low susceptibility to regorafenib. When we integrated NGS sequencing with RT-PCR for EGFRvIII, we observed that patients with mutant MAPK had a significantly worse prognosis than patients with mutations in EGFR. Since the ERK-MAPK pathway considered peculiar of the mesenchymal subtype of GBM tumours, we hypothesised that regorafenib non-responders might harbour the mesenchymal subtype of GBM.

Another step in the direction of identifying molecular predictors of responsiveness to regorafenib was the identification of the association between AMPK (AMP-activated protein kinase) activation and positive clinical response in relapsed GBM patients upon regorafenib treatment (Ref. 53). Starting from observations in preclinical studies, which revealed an association between response to antiangiogenic drugs and the glycolytic activity of tumours or the activity of the liver kinase B1 (LKB1)/AMPK pathway (Refs 54, 55), the authors performed immunohistochemical and digital pathology analyses on specimens derived from relapsed GBM patients, treated or not with regorafenib. On these samples, they characterised a panel of glycolysis- and AMPK-related proteins (the OXPHOS marker monocarboxylate-transporter 1 (MCT1) and the glycolysis-related marker MCT4; phosphorylated AMPK, and a canonical target of AMPK activity, phosphorylated acetyl-CoA carboxylase (pACC)). Their hypothesis was that the antiangiogenic therapy allows an activation of AMPK and this leads to a metabolic reprogramming impairing cell proliferation. Even if this study has been performed on a small cohort of patients, the indication is in line with the hypothesis and confirmed that a positive clinical outcome is linked to an activation of AMPK pathway, more precisely pACC, that might be considered a predictive marker for GBM response to regorafenib.

#### Preclinical in vivo studies

The efficacy of regorafenib in inhibiting tumour vascularisation of a xenograft model of GBM was demonstrated for the first time by Wilhelm *et al.* (Ref. 15) in 2011. Authors demonstrated that regorafenib exerts a huge antiangiogenic effect and a dose-dependent tumour growth inhibition, characterised by significant reduction of tumour cells proliferative index, as addressed by immunostaining with KI67 antibody. The anticancer effectiveness of regorafenib on GBM was confirmed in xenograft models established in immunosuppressed mice upon injection of the GBM stable cell line U87 (Ref. 31). Authors demonstrated a huge size, weight and growth rate of GBM xenografts, accompanied by a reduced cellular proliferation index (as addressed by Ki67 expression) and by an extended mice survival when compared to the control group.

## Expert and topical summary

Clinical observations evidence an improved prognosis in a subset of regorafenib-treated GBM relapsed patients. Since adverse side effects of regorafenib in GBM patients have been recently reported (Refs 56, 57), it will be of key importance to define molecular predictive markers able to allow a stratification of GBM patients, responders versus non-responders, in the perspective of a patient-tailored therapy. We believe that a wide characterisation and identification of molecular key markers of regorafenib responsiveness across this heterogeneous cancer is urgently needed. The achievement of this goal passes through two main steps: (1) understanding of regorafenib molecular mechanisms of action; (2) identification of genes and pathways whose activation or inhibition may overcome regorafenib resistance in non-responders and might increase its activity in responders. Addressing these points will help to uncover molecular biomarkers to predict GBM patients' sensitivity to regorafenib, therefore allowing patients pre-selection for targeted therapy, and the identification of new treatment strategies to overcome drug resistance.
